# Colorectal neoplastic emergencies in immunocompromised patients: preliminary result from the Web-based International Register of Emergency Surgery and Trauma (WIRES-T trial)

**DOI:** 10.1007/s13304-023-01521-8

**Published:** 2023-05-09

**Authors:** Federico Coccolini, Alessio Mazzoni, Camilla Cremonini, Luigi Cobuccio, Marsia Pucciarelli, Guglielmo Vetere, Beatrice Borelli, Silvia Strambi, Serena Musetti, Mario Miccoli, Chiara Cremolini, WIRES-T Study Group, Francesco Salvetti, Paola Fugazzola, Marco Ceresoli, Nita Gabriela Elisa, Andrey Litvin, Eftychios Lostoridis, Ali Yasen Yasen Mohamed Ahmed, Dimitrios Manatakis, Ionut Negoi, Orestis Ioannidis, Mustafa Yener Uzunoglu, Joel Noutakdie Tochie, Nicola Cillara, Gia Tomadze, Miklosh Bala, Arda Isik, Vinicius Cordeiro Fonseca, Giovanni Bellanova, Wagih Ghannam, Omer Yalkin, Fernando Hernandez Garcia, Fatih Altintoprak, Dimitar Hadzhiev, Mircea Chirica, Monica Zese, Dimitros Balalis, Yunfeng Cui, Davide Luppi, Luigi Romeo, Andrea Muratore, Elia Giuseppe Lunghi, Yovtcho Yovtchev, Ioannis Nikolopoulos, Maid Omerovic, Maurizio Zizzo, Lara Ugoletti, Gianluca Costa, Rocco Scalzone, Stefano Perrone, Savino Occhionorelli, Matteo Nardi, Francesca Gubbiotti, Fausto Catena, Ali Muhtaroglu, Rosa Scaramuzzo, Helene Corte, Carlos Yanez, Andee Dzulkarnaen Zakaria, Charalampos Seretis, Roberta Gelmini, Vincenzo Pappalardo, Filippo Paratore, Ruslan Sydorchuk, Francesk Mulita, Yasin Kara, Elena Adelina Toma, Michail Vailas, Maria Sotiropoulou, Fabio Benedetti, Mahamad Elbahnasawy, Maria Grazia Sibilla, Gennaro Martines, Beslen Goksoy, Dimitar Hadzhiev, Dario Parini, Claudia Zaghi, Mauro Podda, Aleksey Osipov, Giuseppe Brisinda, Giovanni Gambino, Lali Akhmeteli, Krstina Doklestic, Zlatibor Loncar, Dusan Micic, Ivana Lešević, Francesca D’Agostino, Ibrahim Umar Garzali, Yaset Caicedo, Lina Marcela, Paola Andrea Gasca Marin, Konstantinos Perivoliotis, Ioannis Ntentas, Arthur Kuptsov, Evgeni Dimitrov, Sharfuddin Chowdhury, Tapan Patel, Massimo Sartelli, Dario Tartaglia, Massimo Chiarugi

**Affiliations:** 1 General, Emergency and Trauma Surgery Department, Pisa University Hospital, Via Paradisa, 2, 56124 Pisa, Italy; 2 Oncology Department, Pisa University Hospital, Pisa, Italy; 3 Statistical Department, Pisa University, Pisa, Italy

**Keywords:** Cancer, Colon, Immunosuppression, Emergency, Surgery, Survival, Mortality, Morbidity, Acute care

## Abstract

Association of advanced age, neoplastic disease and immunocompromission (IC) may lead to surgical emergencies. Few data exist about this topic. Present study reports the preliminary data from the WIRES-T trial about patients managed for colorectal neoplastic emergencies in immunocompromised patients. The required data were taken from a prospective observational international register. The study was approved by the Ethical Committee with approval n. 17575; ClinicalTrials.gov Identifier: NCT03643718. 839 patients were collected; 753 (80.7%) with mild–moderate IC and 86 (10.3%) with severe. Median age was 71.9 years and 73 years, respectively, in the two groups. The causes of mild–moderate IC were reported such malignancy (753–100%), diabetes (103–13.7%), malnutrition (26–3.5%) and uremia (1–0.1%), while severe IC causes were steroids treatment (14–16.3%); neutropenia (7–8.1%), malignancy on chemotherapy (71–82.6%). Preoperative risk classification were reported as follow: mild–moderate: ASA 1–14 (1.9%); ASA 2–202 (26.8%); ASA 3–341 (45.3%); ASA 4–84 (11.2%); ASA 5–7 (0.9%); severe group: ASA 1-1 patient (1.2%); ASA 2–16 patients (18.6%); ASA 3–41 patients (47.7%); ASA 4–19 patients (22.1%); ASA 5–3 patients (3.5%); lastly, ASA score was unavailable for 105 cases (13.9%) in mild–moderate group and in 6 cases (6.9%) in severe group. All the patients enrolled underwent urgent/emergency surgery Damage control approach with open abdomen was adopted in 18 patients. Mortality was 5.1% and 12.8%, respectively, in mild–moderate and severe groups. Long-term survival data: in mild–moderate disease-free survival (median, IQR) is 28 (10–91) and in severe IC, it is 21 (10–94). Overall survival (median, IQR) is 44 (18–99) and 26 (20–90) in mild–moderate and severe, respectively; the same is for post-progression survival (median, IQR) 29 (16–81) and 28, respectively. Univariate and multivariate analyses showed as the only factor influencing mortality in mild–moderate and severe IC is the ASA score. Colorectal neoplastic emergencies in immunocompromised patients are more frequent in elderly. Sigmoid and right colon are the most involved. Emergency surgery is at higher risk of complication and mortality; however, management in dedicated emergency surgery units is necessary to reduce disease burden and to optimize results by combining oncological and acute care principles. This approach may improve outcomes to obtain clinical advantages for patients like those observed in elective scenario. Lastly, damage control approach seems feasible and safe in selected patients.

## Introduction

People with immunocompromised state are increasing due to an augmented number of transplant recipients, extended indications for immunosuppressant medication or chemotherapy, patients with chronic renal failure on hemodialysis and the prevalence of acquired or inherited immunodeficiency. Contemporarily the number of older people is increasing as well. Colorectal cancer is a diffused disease with an incidence progressively increasing with the age. These factors may mix in patients with colorectal neoplastic emergencies (CRNE) associated to an immunocompromised state. Few data exist regarding this topic. Information and indication about this important complication of a diffused disease are needed. Present paper aims to report preliminary data from the Web-based International Register of Emergency Surgery and Trauma (WIRES-T trial). A complete and detailed description of a vast international cohort of patients affected by CRNE associated to immunocompromission (IC) will be presented and analyzed.

## Materials and methods

### Patient selection

Clinical data of patients affected by neoplastic colorectal emergencies in IC patients included in a multicenter international registry (Web-based International Registry of Emergency Surgery and Trauma (WIRES-T)) were analyzed. Patients were divided into two groups: mild–moderate and severe IC according to the definition of mild–moderate and severe IC reported by the recently published guidelines about the topic [1].

### Data analysis

Age, sex, body mass index (BMI), American Society of Anesthesiologists-Physical Status Classification System (ASA), surgical management, surgical approach, WISS (WSES complicated intrabdominal infections Score score), need for intensive care, open abdomen, post-operative complications (Clavien–Dindo), length of stay, in-hospital mortality, and follow-up data (overall survival, disease-free survival, post-progression survival) were accrued into a web-based electronic database. Quantitative parameters were reported as mean and standard deviation for normally distributed data, while non-normally distributed data were described as the median and interquartile range (IQR). Qualitative parameters were reported as absolute numbers and percentages. Shapiro–Wilk test was performed to verify the normality of the quantitative distributions. Correlations between quantitative and/or ordinal data were investigated with Spearman’s rank correlation test. Associations between categorical data were explored with Chi-squared test or Fisher’s exact test. Univariate and multivariate regressions were carried out using generalized linear models (GLM). Statistical analysis was carried out with R 4.0.3 for Windows.

The study was approved by the coordinating center Ethical Committee Pisa University Hospital (EC approval number 17575); ClinicalTrials.gov Identifier: NCT03643718.

## Results

**Fig. 1:**
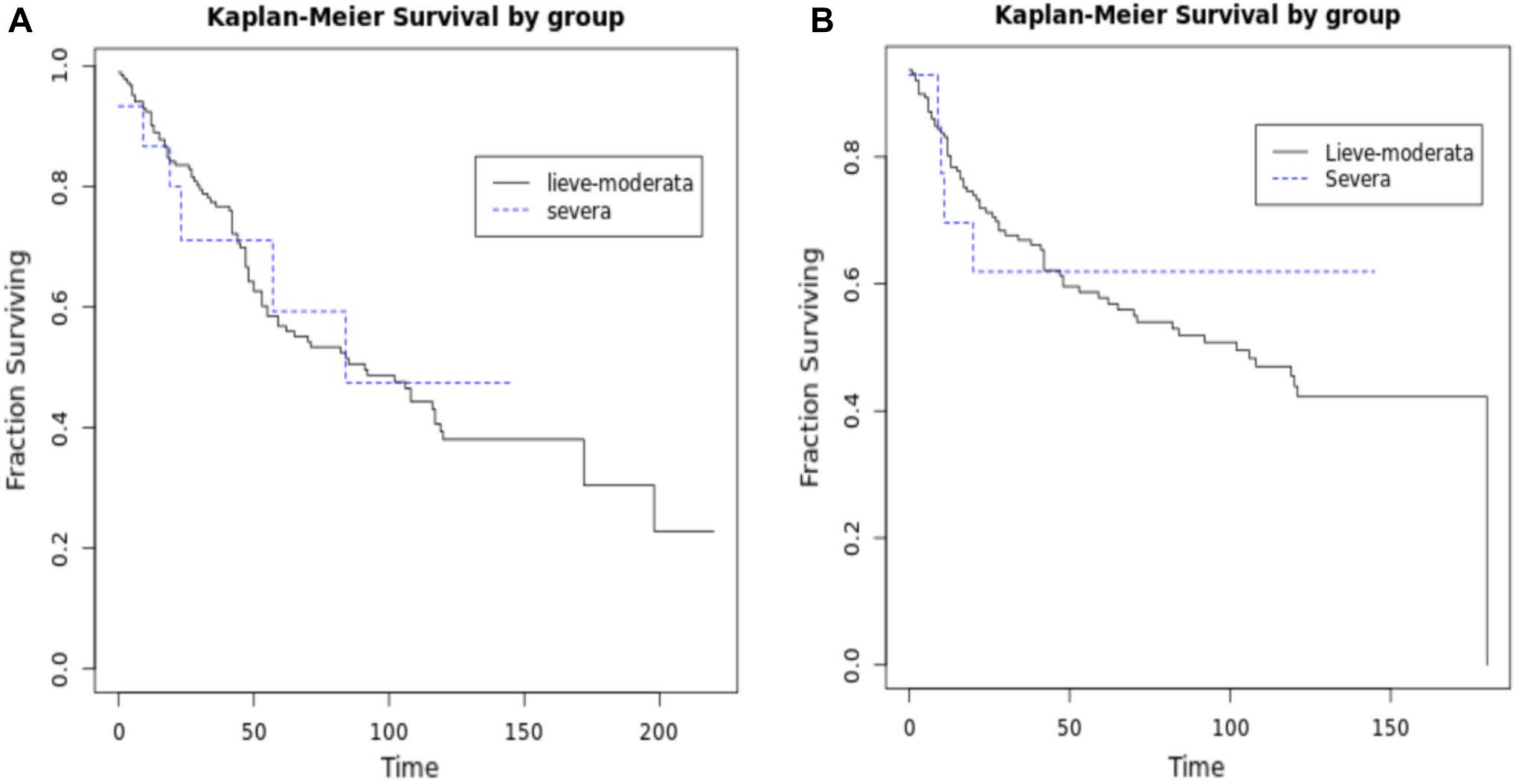
Survival analysis disease-free survival (**A**)—overall survival (**B**)

Present study collected 839 patients; 753 (80.7%) has been classified in mild–moderate group and 86 (10.3%) in severe one (Table 1). The median age was 71.9 years and 73 years, respectively, in mild–moderate and severe group. The causes of mild–moderate IC were reported such malignancy [753 (100%)], diabetes [103 (13.7%)], malnutrition [26 (3.5%)] and uremia [1 (0.1%)], while severe IC causes were steroids treatment [14 (16.3%)]; neutropenia [7 (8.1%)], malignancy on chemotherapy [71 (82.6%)]. Concerning mild–moderate group, combined IC causes were represented in 126 patients: 2 contributing factors in 122 cases and 3 contributing factors in 4 patients; while in severe group were reported 5 patients: in 4 cases (4.7%) has been reported 2 contributing factors while in 1 patient (1.2%) the factors found were 3. Preoperative risk classification were reported as follow: mild–moderate: ASA 1—[14 (1.9%)]; ASA 2—[202 (26.8%)]; ASA 3—[341 (45.3%)], ASA 4—[84 (11.2%)]; ASA 5—[7 (0.9%)]; severe group: ASA 1—1 patient (1.2%), ASA 2—16 patients (18.6%)], ASA 3—41 patients (47.7%), ASA 4—19 patients (22.1%), ASA 5—3 patients (3.5%); lastly, ASA score was missing for 105 cases (13.9%) and 6 cases (6.9%) in mild–moderate and in severe groups, respectively. WISS scores (Median, IQR) are 2 (2–5) and 7 (2–10) in mild–moderate and severe IC groups, respectively. Indications for surgery and the disease site are reported in Table 1. All patient enrolled underwent urgent/emergency surgery according to data reported in Table 2. Damage control approach with open abdomen was adopted in 18 patients with results reported in Table 2. Post-operative complications according to Clavien–Dindo classification were in mild–moderate group: 0—[134 (17.8%)], 1—[255 (33.9%)], 2—[106 (14.1%)], 3—[29 (3.9%)], 4—[16 (2.1%)], 5—[30 (3.9%)], and in severe group: 0—[36 (41.9%)], 1—[16 (18.6%)], 2—[15 (17.4%)], 3—[5 (5.8%)], 4—[3 (3.5%)], 5—[11 (12.8%)]. Median Intensive Care Unit (ICU) and hospital lengths stay were, respectively, 0.8 and 12.5 days in mild–moderate and 1 and 12 days in severe groups. Histology reports are listed in Tables 3, 4. Mortality rates were 5.1% and 12.8% in mild–moderate and severe groups, respectively. The long-term survival data are as follow: in mild–moderate disease-free survival (DFS) (median, IQR) is 28 (10–91) and in severe IC, it is 21 (10–94). Overall survival (OS) (median, IQR) is 44 (18–99) and 26 (20–90) in mild–moderate and severe, respectively; the same is for post-progression survival (PPS) (median, IQR) 29 (16–81) and 28 (17–), respectively (Table 1, Fig. 1).

**Table 1:** Patients’ characteristics

Characteristics	Mild–moderate IC		Characteristics	Severe IC	
	Total	%		Total	%
	753	100		86	100
Age			Age		
< 50	50	6.6	< 50	5	5.8
50–59	75	9.9	50–59	9	10.5
60–69	149	19.8	60–69	16	18.6
70–79	233	30.9	70–79	20	23.3
80–89	205	27.2	80–89	28	32.6
90–100	32	4.2	90–100	6	6.9
Unknown	7	0.9	Unknown	2	2.3
Malignancy	753	100	Steroids	14	16.3
Diabetes	103	13.7	Neutropenia	7	8.1
Malnutrition	26	3.5	Chemotherapy	71	82.6
Uremia	1	0.1	Overlap (2 factors)	4	4.7
Overlap (2 factors)	122	16.2	Overlap (3 factors)	1	1.2
Overlap (3 factors)	4	0.5			
ASA*			ASA		
1	14	1.9	1	1	1.2
2	202	26.8	2	16	18.6
3	341	45.3	3	41	47.7
4	84	11.2	4	19	22.1
5	7	0.9	5	3	3.5
Clavien–Dindo score*			Clavien–Dindo score		
0	134	17.8	0	36	41.9
1	255	33.9	1	16	18.6
2	106	14.1	2	15	17.4
3	29	3.9	3	5	5.8
4	16	2.1	4	3	3.5
LOS (median, IQR)	10 (8–14)		LOS median, IQR)	11 (7–15)	
ICU LOS (median, IQR)	0 (0–0)		ICU LOS (median, IQR)	0 (0–1)	
Mortality*	38	5,1	Mortality#	11	12,8
DFS (median, IQR)	28 (10–91)		DFS (median, IQR)	21 (10–94)	
OS (median, IQR)	44 (18–99)		OS (median, IQR)	26 (20–90)	
PPS (median, IQR)	29 (16–81)		PPS (median, IQR)	28 (17–.)	

*IC* immunocompromission; *LOS* length of stay; *ICU LOS* intensive care unit length of stay; *DFS* disease-free survival; *OS* overall survival, *PPS* post-progression survival

*ASA: missing 105; Clavien–Dindo: missing 183; mortality: missing 38

^#^Mortality: missing 11

**Table 2:** Preoperative characteristics

	IC mild–moderate *N* = 753		IC severe *N* = 86	
	*N*	%	*N*	%
*Diagnosis*				
Occlusion	650	86.2	62	72.1
Occlusion with diastatic perforation	28	4.3	3	4.8
Occlusion and bleeding	0	0	4	4.7
Perforation	119	15.8	16	18.6
Bleeding	32	4.3	1	1.2
Peritonitis	108	14.3	19	22.1
Localized peritonitis	57	52.8	8	42.1
Diffused peritonitis	51	47.2	11	57.9
WISS (median, IQR)	2 (2–5)		7 (2–10)	
*Tumor site*				
Right colon	158	21	26	30.2
Transverse colon	62	8.2	5	5.8
Splenic flexure	48	6.4	5	5.8
Left colon	117	15.5	12	14
Sigmoid colon	304	40.4	28	32.6
Rectum	64	8.5	10	11.6

*IC* immunocompromission; *WISS* WSES cIAIs Score (*IAI* intra-abdominal infections)

**Table 3:** Intra-operative characteristics

	IC mild–moderate *N* = 753		IC severe *N* = 86	
	*N*	%	*N*	%
*Radical surgery*				
Total	701	93.1	71	82.6
Right hemicolectomy	184	26.1	27	38
Left hemicolectomy	175	24.9	14	19.7
Sigmoidectomy	78	11.1	5	7
Ileocecal resection	4	0.6	2	2.8
Anterior rectal resection	51	7.2	1	1.4
Hartmann procedure	97	13.8	11	15.5
Abdomino-perineal resection sec. Miles	4	0.6	1	1.4
Subtotal colectomy	73	10.4	8	11.3
Total colectomy	14	2	2	2.8
Colonic decompression as bridge to surgery	21	3	0	0
*Palliative surgery*				
Total	52	6.9	15	17.4
Colostomy	44	84.6	15	100
Right hemicolectomy	1	1.9	0	0
Hartmann procedure	3	5.8	0	0
By-bass	4	7.7	0	0
*Surgical approach*				
Open	653	86.7	77	89.5
Laparoscopic	100	13.3	9	10.4
Laparoscopic converted to open	28	28	3	33.3
*Damage control—open abdomen*				
Total	15	2	3	3.5
NPWT	9	60	1	33.3
Bogota bag	1	6.7	0	0
Skin closure	3	20	2	66.6
Unknown	2	13.3	0	0
Definitive abdominal (fascial) closure	11	73.3	3	100
Intestinal anastomoses	6	40	1	33.3

*IC* immunocompromission; *NPWT* negative pressure wound therapy

**Table 4:** Histology

	IC mild–moderate *N* = 753		IC severe *N* = 86	
	*N*	%	*N*	%
Adenocarcinoma	331	82.2	35	72.9
Mucinous adenocarcinoma	61	15.2	12	25
Medullar carcinoma	1	0.2	0	0
Poorly differentiated	4	1	0	0
Neuroendocrine	3	0.7	1	2.1
High grade dysplasia adenoma	2	0.5	0	0
Lipoma	1	0.2	0	0
Unknown	350	46.5	38	44.2
Grading				
G1	9	2.7	0	
G2	205	60.5	20	52.6
G3	124	36.6	18	47.4
G4	3	0.3	0	
N/A	411	55	48	55.8

Univariate and multivariate analyses showed as the only factor influencing mortality in mild–moderate and severe IC is the ASA score (Tables 5, 6, 7).

**Table 5:** Univariate analysis: comparison between deceased and survived patients with mild–moderate IC

Total, *N* = 753*	Deceased	Survived	*P* value
	*N* = 38	*N* = 669	
Age	79 (66–84)	73 (64–81)	0.096
Diabetes	4 (10.5%)	94 (14.1%)	0.541
Malnutrition	0	21 (3.1%)	0.268
One cause of IC	34 (89.5%)	556 (83.1%)	0.304
Two causes of IC (2)	4 (10.5%)	111 (16.6%)	0.324
Three causes of IC (3)	0	2 (0.3%)	0.895
ASA**			** < 0.001**
1	0	13 (2.2%)	
2	5 (15.2%)	185 (31.5)	
3	14 (42.4%)	314 (53.5)	
4	9 (27.3%)	73 (12.4%)	
5	5 (15.2%)	2 (0.3%)	

Malignancy and uremia not evaluable in the analysis due to their distribution (malignancy present in every patient, uremia present in only one patient)

Bold values indicate statistically significant results

*Missing 46

**Missing 105

***Missing 183

**Table 6:** Univariate analysis: comparison between deceased and survived patients with severe IC

Total, *N* = 86*	Primary outcome: mortality	Primary outcome: survival	*P* value
	*N* = 10	*N* = 71	
Age	81 (79–90)	72 (61–83)	**0.017**
Age ≥ 70**	8 (100%)	42 (59.2%)	**0.024**
Steroids	1 (10%)	11 (15.5%)	0.543
Neutropenia	0	7 (9.9%)	0.588
Chemotherapy	9 (90%)	59 (83.1%)	0.496
One cause of IC	10 (100%)	66 (93%)	1.000
Two causes of IC (2)	0	4 (5.6%)	1.000
Three causes of IC (3)	0	1 (1.4%)	1.000
ASA***			** < 0.001**
1	0	1 (1.5%)	
2	0	15 (23.1%)	
3	2 (20%)	36 (55.4%)	
4	6 (60%)	13 (20%)	
5	2 (20%)	0	

Bold values indicate statistically significant results

*Missing 5

**Missing 2

***Missing 6

*IC* immunocompromission

**Table 7:** Multivariate analysis for mortality in patients with severe immunocompromission

	Adjusted *p*	OR	95% CI	
Age	0.263	1.057	0.959	1.164
ASA	0.020	7.019	1.364	36.133

Hosmer and Lemeshow Goodness of fit test p 0.250. AUROC = 0.844 (0.720–0.968)

*Binary logistic regression was performed with potentially causative variables, resulted statistically significant at the univariate analysis. Multicollinearity test was check before doing multivariate analysis

## Discussion

IC patients developing CRNE represent an important and under-investigated topic. IC influences the outcomes in patients with CRNE. Age is a recognized risk factor for IC and colon cancer [2–4].

Present data demonstrated a higher incidence of mild–moderate and severe IC during the seventh and the eighth decade. The higher incidence of complication in male gender may be due to the higher incidence of neoplastic disease in males.

The most frequent occurrence site of CRNE is the sigmoid colon with an incidence of 40% and 32.6% in mild–moderate and severe IC, respectively. Even in right colon tumor, IC is linked to a different incidence of emergencies. In mild–moderate IC, they happen in 19.4% and in severe in 30.2% of cases. Descending colon is less involved 15.5% and 14% in mild–moderate and severe, respectively. Chen et al. already published similar data, their right CRNE incidence, however, is 9% [5]. The severity of IC seems to not be related to the site of disease complication. Intestinal occlusion is the most frequent complication, and it happens in 86.2% of patients with mild–moderate and in 72.1% of severe IC. The 4.3–4.8% in mild–moderate and severe IC, respectively, experienced diastatic perforation; previous data reported a higher incidence (12–19%) [6]. This may be explained with the fact that this cohort represents the biggest homogeneous series ever published. For this reason, the percentage may be affected by the case mix and by the sample size. In fact, surgical emergency registries have as one of the main purposes to accrue many data about scattered diffused disease to analyze something closer to the real clinical entity focused on by the specific research.

Intestinal perforation rate is 15.8% in mild–moderate and 18.6% in severe IC patients and this is higher than the one reported in previous articles (2.6–12.6%). This may be due to the more rapid evolution of the disease or to the milder symptoms presentation leading to a delayed diagnosis. This may potentially influence the diagnostic and therapeutical pathway.

According to the literature, CRNE may express with massive bleeding in 8–26% of cases [7]. Present study reported a massive bleeding incidence of 4.3%. This percentage increases up to 5.8% in those cases where bleeding is associated to occlusion.

Surgical approach showed a preference for laparotomic interventions. In fact, the 86.7% of patients experienced laparotomic approach and the 9.6% the laparoscopic one. Conversion rate from laparoscopic to laparotomic is 3.7%. This is due to the emergency situations and to the diffused limited use of laparoscopy in emergency setting [8].

As a counterpart, laparoscopy in emergency setting in perforated patients with diffused peritonitis and potential serious metabolic derangements, represents a great challenge. It should be reserved to selected patients in specialized emergency surgery centers with all the necessary facilities and expertise. Damage control approach is feasible even in the case of CRNE. No univocal indication to the use of this approach in IC patient exists [8, 9].

In this big cohort of patients, a few (18 patients) experienced open abdomen procedures with good results in terms of survival, abdominal definitive closure, and intestinal anastomosis as showed in Table 3. Among the mild–moderate IC and severe IC patients 73.3% and 100%, respectively, experienced definitive fascial closure after OA procedures. In the two groups 40% and 33.3% of the patients experienced intestinal anastomosis with no reported complications. Open abdomen following a damage control procedure, in fact, may represent a potential bridge solution to allow patients recovering from severe physiological derangements before being undergone to definitive surgical procedure. Damage control approach is usually reserved only to extremely sick patients. In fact, these 18 patients in need for damage control procedures may have not overcome the stress of complex and oncological adequate surgical procedures due to the severe physiological derangements. A step approach as the damage control one allowed them to be treated and to survive safely. These data about the potential benefit of damage control approach in CRNE represent an interesting result and something that would need more specific studies and evaluation. Risk–benefit balance in these patients must be accurately evaluated to prevent futile surgical procedure. Accurate patient selection is mandatory to avoid too sick patients with very limited physiological reservoir undergoing open abdomen procedures. This may not only lead to negative outcome and may also increase the management burden on the patients without effective results. Moreover, the economical counterpart of damage control procedures with complex and expensive approaches in patients that likely will not obtain any benefit must be accurately evaluated. Lastly, the definitive closure intervention in OA patients may represent a complex surgical intervention due to several factors as adhesions or technical issues; for this reason too, accurate selection of patients is necessary.

Hospital stay is generally longer in complicated patients. Immunocompromised and neoplastic patients are by definition frail and exposed to higher complication risk [10]. Their management is very difficult and must be multidisciplinary. Present study reported a percentage of uneventful admission of 41.9%. The post-operative course data are reported in almost 80% of patients. They showed that when these patients are treated in equipped and experienced centers by dedicated personnel, they may beneficiate of good outcomes [11, 12]. Emergency and frail patients should be centralized to specific centers with the necessary facilities and expertise. With this strategy, the best outcome possible can be achieved.

Post-operative mortality rate in mild–moderate IC was 4% and in severe 12.8%. Reported mortality is 5–34% [12–15].

Management of IC patients is difficult especially in emergency setting where physiological derangements and infections are associated to the surgical emergency. The present cohort’s lower mortality rate compared to the literature is probably due to the management done by dedicated emergency general surgical units. Dedicated emergency general surgery teams in fact are trained to manage surgical emergency in frail and sick patients because they are prone to multifactorial interpretation of each single case, to balance the treatment, and to manage patients with a multidisciplinary approach keeping into consideration not only the anatomical variables but also the physiological ones.

Survival in oncologic patients is strictly related to the oncological radicality in performing surgical procedures. Present data showed as notwithstanding the emergency setting, oncologic accurate resections was obtained in the most part of cases with a number of retrieved lymph nodes higher of 12 in up to 81.6% of cases. The necessity to balance the surgical appropriateness of intervention and the emergency setting is sometimes underestimated. In such a complex cohort of patients, however, the emergency general surgeon may be the most appropriate in combining anatomical and physiological necessities. Oncologic surgery principles must be respected together with the acute care and emergency ones.

Advanced disease (T4 tumor) in mild–moderate IC was discovered in 13.6% of cases and metastasis was present in 16.8% of patients. In severe IC, advanced disease was present in 7.5% and metastasis in 34.4% of patients. Baer et al. reported an advanced disease incidence up to 38% of cases [16]. Disease stage is more advanced in severe IC than in mild–moderate. In fact, in the first group, it is more represented the 4th stage and in the second the 3rd stage. This is probably due to the more aggressive behavior of the cancer in patients with less effective immunological answer [17, 18].

The 5-year survival in the literature is reported to be 39%. In present study, it was 55.8% and 60% in mild–moderate and severe IC, respectively, reflecting the necessity to manage these patients in referral centers in dedicated emergency surgery unit to maximize the gain for the patients. No statistical differences, however were found at the survival analysis between the two groups (Fig. 1).

The main strength of the present study is its multicentricity and the accrual of data from high-volume emergency general surgery units around the world. Therefore, it may represent a clinical scenario reflecting the real clinical incidence of this surgical emergency.

The second strength of the study is the numerosity of the sample. It represents the biggest ever published cohort of homogeneous patients affected by this disease.

The main limitation is that the prospective observational design cannot warrant that all patients included are all those really managed inside each surgical unit. It should be supposed, however, that scientific honesty of the different researchers may help in overcoming this potential bias and may warrant that all positive and negative outcomes are included into the register.

## Conclusion

Colorectal neoplastic emergencies in immunocompromised patients are more frequent in elderly. Sigmoid and right colon are the most involved. Emergency surgery is at higher risk of complication and mortality; however, management in dedicated emergency surgery units is necessary to reduce disease burden and to optimize results by combining oncological and acute care principles. This approach may improve outcomes to obtain clinical advantages for patients like those observed in elective scenario. Lastly, damage control approach seems feasible and safe in selected patients.

## Data Availability

Data cannot be shared openly, to protect study participant privacy. They may be available contacting the main author of the paper.
